# Supplementary material for: Association between oral microbiome and seven types of cancers in East Asian population: a two-sample Mendelian randomization analysis
Source: Front Mol Biosci. 2023 Nov 21;10:1327893. doi: 10.3389/fmolb.2023.1327893 (PMC10702768; doi:10.3389/fmolb.2023.1327893)
Supplement: Supplementary file 1 [file DataSheet1.ZIP › Supplementary Materials/MR plots for tongue/tongue═╝/Pancreatic cancer/╥╚╧┘░⌐_╔╕╤í╜ß╣√/pheno.2249_to_pancreatic cancer_leave_one_out.pdf]

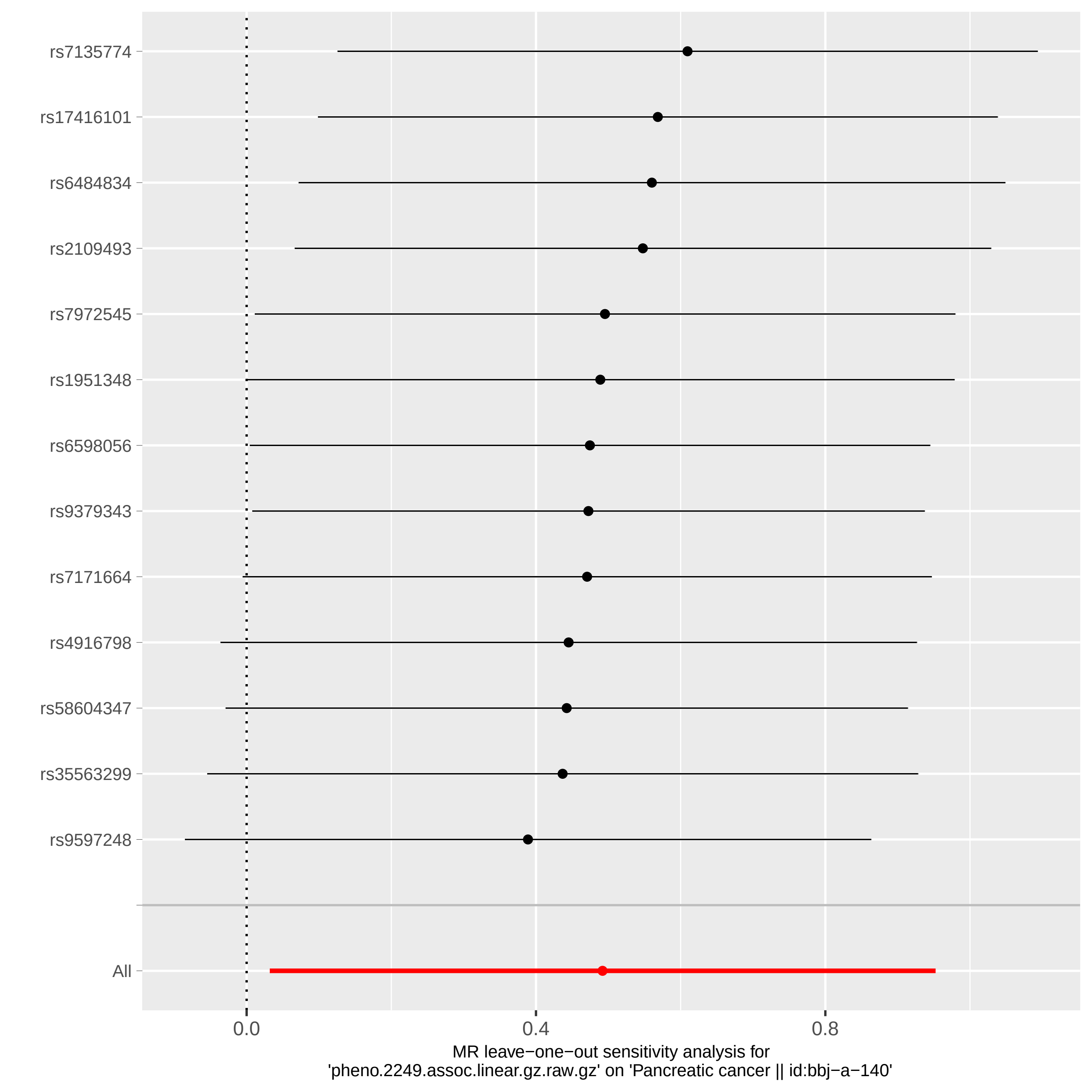
rs7135774
rs17416101
rs6484834
rs2109493
rs7972545
rs1951348
rs6598056
rs9379343
rs7171664
rs4916798
rs58604347
rs35563299
rs9597248
All
0.0
0.4
0.8
MR leave−one−out sensitivity analysis for
'pheno.2249.assoc.linear.gz.raw.gz' on 'Pancreatic cancer || id:bbj−a−140'
